# Physicochemical responses of soil and caraway crop to drip irrigation with magnetized saline irrigation

**DOI:** 10.1038/s41598-026-45468-3

**Published:** 2026-05-26

**Authors:** Nessrien S. Abd Elkareem, Alia Amer, Aml Shahin, Mohamed A. M. Moursy

**Affiliations:** 1 Water Management Research Institute, National Water Research Center, El-Qanater El-Khairiya, Egypt; 2 Medicinal and Aromatic Plants Research Department, Horticulture Research Institute, Agricultural Research Center, Giza, Egypt

**Keywords:** Caraway, Magnetic, Surface Drip, Subsurface Drip, Fruit Yield, Oil Yield, Engineering, Environmental sciences, Hydrology, Plant sciences

## Abstract

Magnetization of irrigation water has gained increasing attention in agriculture. Salinity stress markedly decreases caraway growth and productivity. This study aimed to determine the optimal irrigation approach using magnetic water technology for improving caraway genotype efficiency, yield, and irrigation water productivity (IWP). This investigation was conducted at the Wadi El-Natrun Research Station, under the auspices of the Water Management Research Institute, National Water Research Center, Egypt. This study included four treatments, using magnetic and non-magnetic water, applied under two different drip irrigation systems (surface and subsurface). The findings indicated that in the absence of magnetic treatment, EC values increased after irrigation at all soil depths. In contrast, the use of magnetized water consistently reduced irrigation requirements in both surface (SDI) and subsurface (SSDI) drip systems, with reductions of 619 and 681 m³/ha, respectively. Furthermore, magnetized water increased IWP by approximately 16% and 14% under SDI in the 1st and 2nd seasons, respectively, compared to non-magnetized treatments. SSDI with magnetized water also had a pronounced positive effect on overall caraway productivity per feddan, with average friut yield increasing by approximately 10.38% and essential oil yield rising by more than 24.56%. The benefit–cost ratio improved from 2.48 to 2.53 under SDI and from 2.54 to 2.62 under SSDI in the first season, highlighting increased economic efficiency. These results underscore the potential of advanced magnetic technologies and modern irrigation practices in enhancing caraway performance under saline stress. Future studies will assess different magnetic field strengths, optimal exposure time, and the best placement of the magnetization unit to maximize water use efficiency.

## Introduction

Water scarcity is a critical global challenge, particularly in arid and semi-arid regions. In Egypt, limited water resources face growing pressure from population growth, climate change, and agricultural expansion, all reliant on the Nile River, with additional concerns from the Grand Ethiopian Renaissance Dam. Addressing this scarcity is a strategic priority to ensure sustainable agriculture and efficient resource management. To respond, Egypt has promoted non-traditional crops, especially medicinal and aromatic plants, to optimize water use, support agricultural development, and increase foreign exchange earnings. Despite their economic importance, production growth remains below global demand, declining slightly by 0.15%, while Egyptian exports maintain competitive market access^1^. Currently, medicinal and aromatic plants occupy only 0.7% of Egypt’s cultivated area^2^. In order to harness their full potential, the Ministry of Agriculture intends to expand cultivation. Therefore, developing integrated and innovative strategies is essential to increase productivity, enhance export competitiveness, and ensure sustainable water use in arid and semi-arid regions^3,4^.

The quality and composition of irrigation water play a crucial role in determining soil salinity levels, which directly affect plant growth and crop productivity^5,6^. Salinity and drought represents one of the most significant abiotic stresses limiting the growth and yield of medicinal plants^7^. Agriculture on reclaimed lands has become increasingly important for enhancing food security; however, these areas are often constrained by poor soil structure and extremely low organic matter content. Such conditions reduce soil fertility, water-holding capacity, and nutrient availability, thereby hindering sustainable crop production (Basak et al.,^8,9^;. To overcome these challenges, integrated soil and water management strategies are essential. Adopting modern irrigation techniques, such as surface and subsurface drip irrigation, is an effective approach to mitigate the negative impacts of saline water on soil and crop growth. These systems reduce surface evaporation and salt accumulation, thereby preserving soil health and optimizing water use^10^. Drip irrigation can significantly decreased soil salinity in the root zone by 37.7%^11^. By enhancing water distribution and irrigation efficiency, these practices improve soil productivity and support the sustainable cultivation of medicinal and aromatic plants under saline and reclaimed soil conditions. Therefore, the implementation of modern irrigation systems is a key component of integrated water and soil management, particularly in newly reclaimed areas where water scarcity and soil salinity are major constraints to agricultural production. This conclusion is strongly supported by field experiments, which reported an 81.6% increase in eggplant yield, a 51.3% improvement in vegetative growth, and a 35% reduction in soil salinity, in addition to enhanced emitter performance and reduced soil resistance^12^.

Magnetic treatment is a recent and promising technology for improving irrigation water quality, particularly for saline and brackish water, while enhancing the management of non-conventional water resources (Abd El-Hady et al., 2024 and Talat Rashad, 2022)^13,14^. It modifies the chemical properties of water, reducing soil salt accumulation and promoting plant growth. Pretreatment with a static magnetic field (MF) improves photosynthesis and reduces total soluble solids and electrical conductivity^15,16^. Similarly, Hilal et al.^17^, demonstrated that magnetic treatment improves the physical properties of water, such as reducing surface tension and increasing water infiltration into the soil, which enhances salt leaching and reduces accumulation around the root zone. Move to the introduction part. Studies show that lettuce irrigated with magnetically treated water produced more leaves and biomass, with better nutrient availability and enhanced salt leaching^18^ and Sary, 2021), while zucchini seeds exhibited higher germination, lower Na⁺ and Cl⁻, and increased soil potassium^19^. Combined with optimized fertilization, magnetic irrigation enhanced crop quality and yield, for instance, crisphead lettuce achieved maximum vegetative growth and yield while reducing production costs by 50% (Abd El-Hady et al., 2024)^13^. Additionally, it improved soil properties such as pH and EC^20^ and altered water characteristics to mitigate salinity stress even under low magnetic fields^21^. For medicinal and aromatic plants like rosemary, magnetic irrigation improved growth, essential oil content, and chemical composition under salinity stress^22^. Several studies show that magnetic irrigation improves soil chemical properties. Amer et al.^23^, found that it reduced soil salinity (ECe), sodium adsorption ratio (SAR), Na⁺, Cl⁻, and SO₄²⁻, while increasing Ca²⁺, Mg²⁺, and K⁺ concentrations. Similarly, Fanous et al.^24^, showed that magnetic treated water (MTW) lowered soil salinity from 4.88 to 6.15 to 1.45–2.83 dS·m⁻¹ over eight months, slightly reduced pH from 8.28 to 8.30 to 7.90–8.05, and decreased NaCl and MgCl₂ levels. Kamel et al.^25^, also reported reductions in soil electrical conductivity (EC) by 3.8% and 7.9% over two consecutive seasons compared to the control. Overall, magnetic water treatment provides a sustainable, environmentally friendly, and effective approach to enhance crop productivity, soil health, and water management under saline and brackish water^12,24^.

Caraway (Carum carvi L.) is a biennial aromatic herb of the Apiaceae family, native to Western Asia, Europe, and Northern Africa, and widely cultivated in Upper Egypt ( Aćimović et al., 2014^26^;. Its seeds and essential oil are used in the food and pharmaceutical industries for their carminative, antispasmodic, and digestive properties, while the leaves are edible^27,28^. Proper treatment and storage of fruits are essential to preserve their bioactive compounds for medicinal and industrial use^26^.

The current study addresses a critical research gap in the cultivation of medicinal and aromatic plants under saline conditions in arid and semi-arid regions. Despite their high economic value and increasing global demand for natural pharmaceutical products, the expansion of these crops is constrained by environmental limitations, particularly soil and water salinity, which severely reduce yield and quality. While most species are sensitive to salinity, a few resilient plants, such as jojoba and cactus, can tolerate stress, whereas high-value crops like hibiscus, caraway, and chamomile require non-saline conditions for optimal growth. In Egypt, cultivated areas remain limited despite the suitability of newly reclaimed lands, reflecting the absence of scientifically based strategies adapted to harsh environments. Advanced irrigation technologies, including magnetic water treatment, have demonstrated potential to alleviate salinity stress and enhance water use efficiency; however, field-based evidence supporting their effectiveness remains scarce.

In response to this challenge, the present study aimed to investigate the effectiveness of magnetic water treatment and modern irrigation strategies in improving the growth, yield, and physiological performance of caraway (Carum carvi L.) under saline stress. Specifically, the research focused on assessing the effects of magnetic water on plant growth, yield components, and essential oil quality, while comparing surface and subsurface drip irrigation systems. Additionally, the study evaluated water use efficiency and examined changes in soil chemical properties induced by magnetically treated irrigation. By integrating these approaches, this study seeks to establish innovative, science-based strategies for the sustainable cultivation of medicinal and aromatic plants in saline-prone regions, emphasizing the role of advanced irrigation techniques and water quality management in optimizing crop performance and resource efficiency.

## Materials and methods

### Study site description

The present study was conducted at the Wadi El-Natrun Research Station, affiliated with the Water Management Research Institute, Agricultural Research Center (ARC), Egypt. The station is situated in Wadi El-Natrun City, El-Beheira Governorate, along latitude 30°24′ N and longitude 30°30′ E, approximately 106 km northwest of Cairo. The region is characterized by an arid climate with low annual rainfall, high temperature fluctuations, and sandy soils with limited fertility conditions, representative of the newly reclaimed desert areas of northern Egypt. This site was selected as an ideal location for evaluating the performance of caraway plants under saline irrigation conditions and assessing the potential of innovative water management techniques, such as magnetic water treatment and advanced drip irrigation systems, in enhancing crop productivity and soil quality.

Prior to cultivation, composite soil samples were collected from various representative locations within the experimental area at two depths (0–20 cm and 20–40 cm) to evaluate the chemical properties of the soil, as shown in Table 1. This analysis represents a crucial step in agricultural research, as it provides essential information about soil texture, water-holding capacity, salinity levels, and pH, key parameters that determine the suitability of soil for cultivating medicinal and aromatic plants, which are generally sensitive to salinity and alkalinity stress. Establishing a baseline characterization of the soil also allows for monitoring potential changes resulting from the application of different irrigation techniques, particularly magnetic water treatment, thereby providing a solid scientific basis for improving soil and water management practices and enhancing crop productivity and quality. Preliminary analyses indicated that the soil at the Wadi El-Natrun Research Station is sandy in texture, with an average sand content of approximately 93% with field capacity 9.13, wilting point 3.14 and a pH ranging between 7.8 and 8.1, classifying it as moderately alkaline. Soil pH is among the most critical factors influencing crop growth and is highly responsive to irrigation water quality, especially when magnetized water is applied. To assess the long-term impacts of irrigation and magnetic water treatment, additional soil samples were collected at the end of the experiment to identify any potential changes in soil chemical and physical properties. Furthermore, groundwater was identified as the primary source of irrigation water at the Wadi El-Natrun Research Station. Water used for irrigation was drawn from a deep well, with a salinity level of approximately 4,300 ppm, as determined by laboratory analysis of water samples. The chemical characteristics of the irrigation water, presented in Table 2, provide a detailed overview of the water quality parameters that may influence soil properties and crop performance under the experimental conditions.

**Table 1:** Soil chemical properties.

No.	pH	EC. (ds.m^− 1^)	Cations(mg/kg)				Anions (mg/kg)		
			Na	K	Ca^2+^	Mg^2+^	Cl	CO_3_	HCO_3_
1	7.76	1.628	844.5	181.5	380	34.2	1364.98	0	170.8
2	7.33	0.934	223	41.5	380	24	282.225	0	88.45
3	7.45	1.603	599.5	152	398	27.6	1153.75	0	94.55
4	7.73	0.857	351.5	78	335	26.4	186.375	0	67.1

**Table 2:** Water chemical properties.

pH	SAR	EC. (ds.m^− 1^)	Cations(mmol/L)				Anions (mmol/L)		
			Na	K	Ca^2+^	Mg^2+^	Cl	CO_3_	HCO_3_
7.65	9.9	6.68	36.96	0.9	9.2	4.75	31.58	----	3.19

### Crop

The experimental field was prepared for caraway (*Carum carvi* L.) cultivation using standard agronomic practices, including plowing, soil leveling. Caraway (*Carum carvi* L.) seeds were obtained from the medicinal and aromatic plant department, Horticulture Research Institute, Agricultural Research Center, Giza, Egypt. According to the Ministry of Agriculture’s recommendations (2005), 47.8 m³ of organic manure per hectare was incorporated during soil preparation, followed by the application of 478 kg/hectare superphosphate (15% P₂O₅). Ammonium sulfate (20.6% N) was applied at 358.5 kg/hectare in two equal splits after seedling establishment and at flowering, while 239 kg/hectare potassium sulfate (48% K₂O) was applied during the early vegetative stage. Foliar sprays of Fe (100 ppm), Zn (50 ppm), and Mn (50 ppm) were applied twice, at 45 and 75 days after sowing, to enhance vegetative growth and essential oil synthesis. Pest control was conducted using recommended pesticides. In the 1 st growing season (2023), sowing was carried out on 10th November, and the crop was harvested on 25th May. In the 2nd season (2024), sowing and harvesting occurred on 12th November and 29th May, respectively. In both seasons, two seeds were sown per hill, with 30 cm spacing between hills and 60 cm between ridges.

### Irrigation systems

**Fig. 1:**
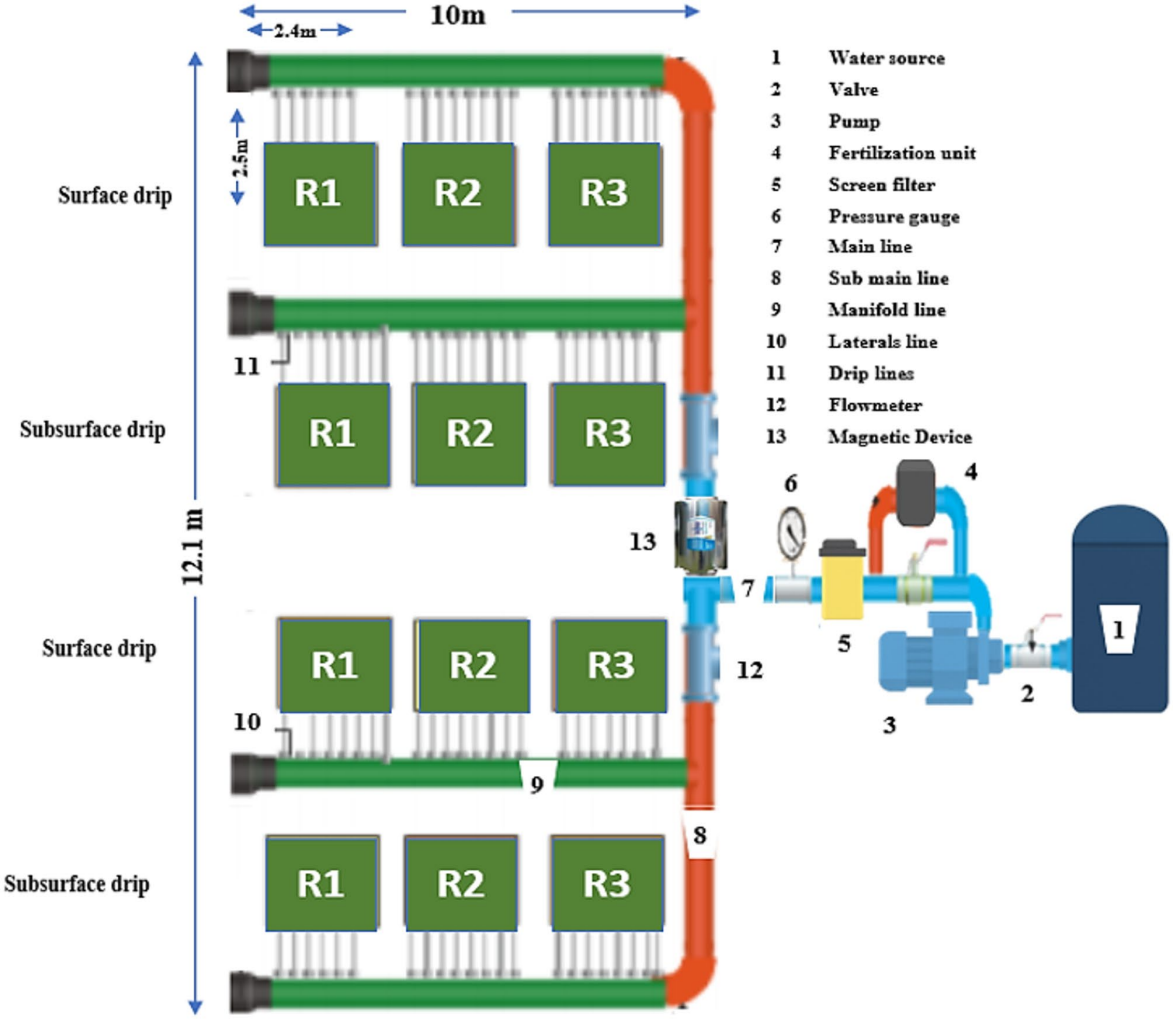
Experimental layout.

To achieve precise control over water quantity and distribution, both surface and subsurface drip irrigation systems were employed in the experiment, showed Fig. 1. The main lines had a diameter of 90 mm, the sub-main lines 75 mm, the manifold lines 50 mm, and the s laterals lines 32 mm. In both systems, drip lines with an outer diameter of 16 mm, fitted with internal GR emitters, each providing 4 L/h at 1.2 bar and spaced 25 cm apart, were employed. The subsurface drip lines were installed at a depth of 15 cm below the soil surface. The irrigation system was designed to ensure uniform water delivery to each plant, thereby enhancing irrigation efficiency and promoting optimal crop growth and yield.

### Magnetic device

The magnetization device used in this study was manufactured by Delta Water Co. It is made of stainless steel with a 2-inch inner diameter, 85 cm length, and a weight of approximately 11 kg. Under these conditions, the duration of exposure of the water to the magnetic field was approximately 0.5 s. The device’s technical specifications, a flow rate of up to 25 m³/h, a pressure of 15 bar, and a temperature of up to 100 °C, with an effective performance for water salinity up to 8000 ppm. It generates a magnetic field of 14,500 Gauss (1.45 Tesla) that alters the physical properties of water as it passes through. Under experimental conditions, exposure of low-salinity water to a magnetic field has been reported to influence the hydrogen-bonding structure, decrease the size of water molecular clusters, and enhance ionic mobility, without altering the EC, as documented in previous studies^29,30^.

### Experimental treatment and condition

The experiment was conducted during two consecutive growing seasons (2023/2024 and 2024/2025) using a split-plot design with three replications to evaluate the effects of magnetized treatments and irrigation system irrigation water on the growth and yield of caraway (*Carum carvi* L.) under field conditions. The main plots were assigned to the irrigation water type, namely magnetized (W₁) and unmagnetized (W₀), while the subplots represented the water irrigation systems, consisting of (SDI) surface drip irrigation (S₁) and (SSDI) subsurface drip irrigation (S₀). The experiment included 12 experimental plots, each with an area of 6 m², separated by 1 m buffer zones to prevent lateral water movement, giving a total experimental area of 121 m². Treatments were arranged randomly within each replication according to the principles of the split-plot design.

### Measurements and calculations

**Soil chemical croperties including**Soil pH, electrical conductivity and sodium adsorption ratio were measured both before sowing and after harvesting the crop, following the standard procedures outlined by Singh et al.^31^,.

### Comprehensive analysis of water studies

#### The actual irrigation requirements or total water use

The actual irrigation requirements under each irrigation system were calculated according to the equation as cited from Morad (2012):

$$\:\mathrm{I}\mathrm{R}\mathrm{a}=\frac{\lceil\:\left({{\uptheta\:}}_{\mathrm{F}\mathrm{C}}-{{\uptheta\:}}_{\mathrm{v}}\right)\times\:\mathrm{B}\mathrm{d}\times\:\mathrm{d}+\rceil\mathrm{L}\mathrm{f}}{\mathrm{E}\mathrm{s}}$$

The actual irrigation requirements (IRa), expressed in mm per interval, were calculated by considering various soil and system parameters. These include system efficiency (Es, %), soil moisture content at field capacity (θFC, %), bulk density (Bd, g/cm³), and the existing soil moisture content under field conditions (θv, %), along with the depth of the soil layer (d). Additionally, the leaching factor (Lf) under the applied irrigation methods was determined using the following equation^32^:

$$\:\mathrm{L}\mathrm{f}=\frac{\mathrm{E}\mathrm{C}\mathrm{w}}{2\mathrm{max}\mathrm{E}\mathrm{c}\mathrm{e}}$$

ECw refers to the salinity of the irrigation water (dS/m), while ECe denotes the average soil salinity tolerated by the crop, measured from a soil saturation extract (dS/m).

The soil moisture content was determined using an electronic sensor (Delta-T Devices, Profile Probe PR2, England) at a depth of up to 30 cm. Irrigation water amounts were determined according to the soil moisture content measured immediately before irrigation.

#### Irrigation water productivity, (IWP, kg/m^3^)

IWP, kg/m³, was endorsed by Pereira et al.^33^, as a modified indicator of water use efficiency.

$$\:\mathrm{I}\mathrm{W}\mathrm{P}\:\:=\frac{\:\:\:\:\:\:\:\:\:\:\:\:\mathrm{Y}\mathrm{a}(\mathrm{k}\mathrm{g}/\mathrm{h}\mathrm{a})\:\:\:\:\:\:\:\:\:\:\:\:\:\:\:\:\:\:\:\:\:\:\:}{\mathrm{A}\mathrm{T}\mathrm{W}\mathrm{U}\:({\mathrm{m}}^{3}/\mathrm{h}\mathrm{a})},\:\mathrm{k}\mathrm{g}/{\mathrm{m}}^{3}$$

Irrigation Water Productivity (IWP), expressed in kg/m³, is calculated as the ratio of total yield (Ya), measured in kilograms per hectar (kg/ha) for either fruit or oil, to the total water use (ATWU), measured in cubic meters per feddan per season (m³/ha/season).

### Crop characteristics

#### Growth parameters and fruit yield measurements

To prevent fruit dispersal, the caraway fruits were picked early in the morning when they were fully ripe with a sickle. After ten days of drying in a well-ventilated, shaded environment, the collected samples were gently threshed to prevent breaking. Measurements were made of the height of the plant, the number of branches per plant, and the number of umbels per plant. Yield metrics collected in this trial were fruit yield/plant (g), weight of 1000 fruits (g), and fruit yield/ha (kg).

#### Chemical analysis of fruit essential oil and its components

As indicated by the Egyptian Pharmacopoeia (1984), the oil was extracted using the hydro-distillation method in a Clevenger apparatus in order to determine the percentage of the essential oil (%). According to Guenther (1961), 100 g of caraway fruits were crushed and then hydro-distilled for an average of three hours. The ratio of the weight of fruits used to the volume of oil extracted was used to compute the essential oil production. Before analysis, the recovered essential oil was kept in a small opaque flask at 4 °C in a dark location after being dehydrated by anhydrous sodium sulfate.

The essential oil was assessed using gas chromatography (Agilent 8890 GC system) and a mass spectrometer (Agilent 5977B GC/MSD) at the Central Laboratory, Institute of Food Industry and Nutrition, National Research Center, Cairo, Egypt. Hexane was used to dilute the samples (1:19, v/v). The GC was equipped with an HP-5 MS column, which measured 30 m by 0.25 m in internal diameter and 0.25 m in film thickness. The following temperature program, a split ratio of 20:1, an injection volume of 2 µl, and a carrier gas (helium) flow rate of 1 ml min-1 were used to complete the analysis: 0 min at 50 °C; 4 min at −1 °C to 240 °C and held for 0 min; 10 min at a rate of 10 °C/min to 280 °C and kept for 5 min. At 280 °C, the injector and detector were maintained. Mass spectra were obtained using electron ionization (EI) at 70 eV, with a solvent delay of 5 min and a spectral range of m/z 40,550. To identify various constituents, the spectrum fragmentation pattern was compared with those found in Wiley and NIST Mass Spectral Library data.

### Economic evaluation

An economic evaluation was conducted to assess the profitability of various treatments. The net return was calculated by deducting the total production costs from the gross income generated by the caraway fruit yield. Water costs for each irrigation treatment were determined by multiplying the unit cost of water by the total volume of irrigation water required for the caraway crop. Furthermore, the cost of the magnetic device was included in the analysis. Additional production expenses such as labor (land preparation, seed procurement, sowing, weeding, fertilizer application, spraying, and harvesting), agrochemicals (including insecticides and pesticides), and the components of the drip irrigation system (comprising low density polyethylene pipes for main lines, sub-mains, and laterals; filters; fertilizer unit; pressure gauges; control valves; water meters; drippers; and other accessories) were all calculated based on the depreciation cost of the entire drip irrigation setup^34^.

### Statistical analysis

Data were analyzed using analysis of variance (ANOVA) with the SPSS statistical package to determine the effects of the main and interaction factors, with significance tested at *p* ≤ 0.05. Mean comparisons were performed using Duncan’s Multiple Range Test (DMRT), and the coefficient of variation (CV%) was calculated to assess the experimental precision and data reliability.

## Results

The following section presents the discussion of the results:

### Effect of different treatments on some soil chemical properties

#### Soil pH

The results of post-experiment analyses revealed that soil pH values ranged between 7.39 and 7.51, all within the slightly alkaline range, with very small and statistically insignificant differences. These findings indicate that magnetic treatment of irrigation water did not have a noticeable effect on soil pH, as the values remained stable without significant variations after applying the different irrigation systems.

#### Electrical conductivity

**Fig. 2:**
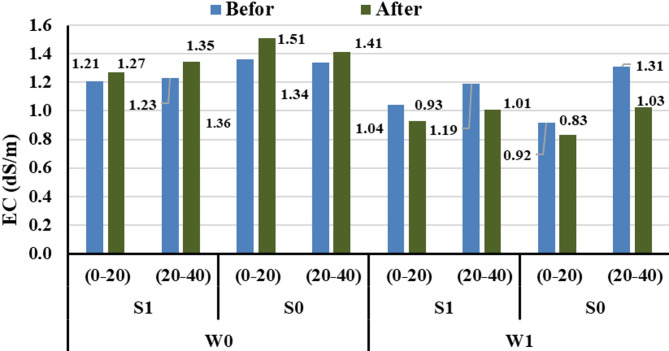
The variation in soil electrical conductivity values under different treatments.

Figure 2 presents the average results of soil chemical analyses across the two study seasons, particularly soil salinity (EC), and the influence of irrigation systems and magnetically treated water on it. The results showed that in the absence of magnetic treatment, EC values increased after irrigation at all soil depths. Specifically, EC rose from 1.358 to 1.420 dS/m in the 0–20 cm layer (a 4.56% increase) and from 1.568 to 1.686 dS/m in the 20–40 cm layer (a 7.53% increase), indicating salt accumulation in the root zone. This increase was more pronounced under subsurface drip irrigation, due to salt concentration near the roots and reduced evaporation losses from the soil surface. In contrast, when the magnetic device was used, EC values either decreased or remained stable after irrigation.

#### Exchangeable sodium percentage

**Fig. 3:**
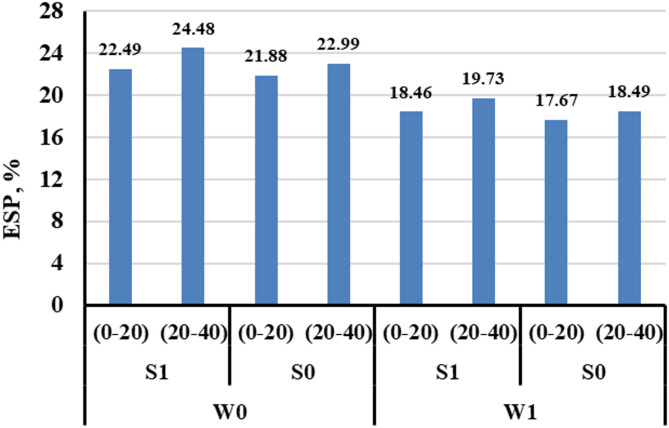
The variation in soil ESP values of the soil post-harvest under different treatments.

The results in Fig. 3 indicated that the exchangeable sodium percentage (ESP) in the soil was significantly influenced by both the irrigation system and the use of magnetic water treatment. Under non-magnetic conditions, ESP values of the soil post-harvest ranged from 21.88% to 24.48%, with slightly higher values observed in the 20–40 cm soil layer compared to the 0–20 cm layer. When magnetic treatment was applied, ESP values decreased across all treatments, ranging from 17.67% to 19.73%, indicating a notable reduction in sodium accumulation. This effect was observed in both drip and subsurface irrigation systems, with the subsurface system showing slightly lower ESP values under magnetic treatment.

### Comprehensive analysis of water studies

#### The actual irrigation requirements

**Fig. 4:**
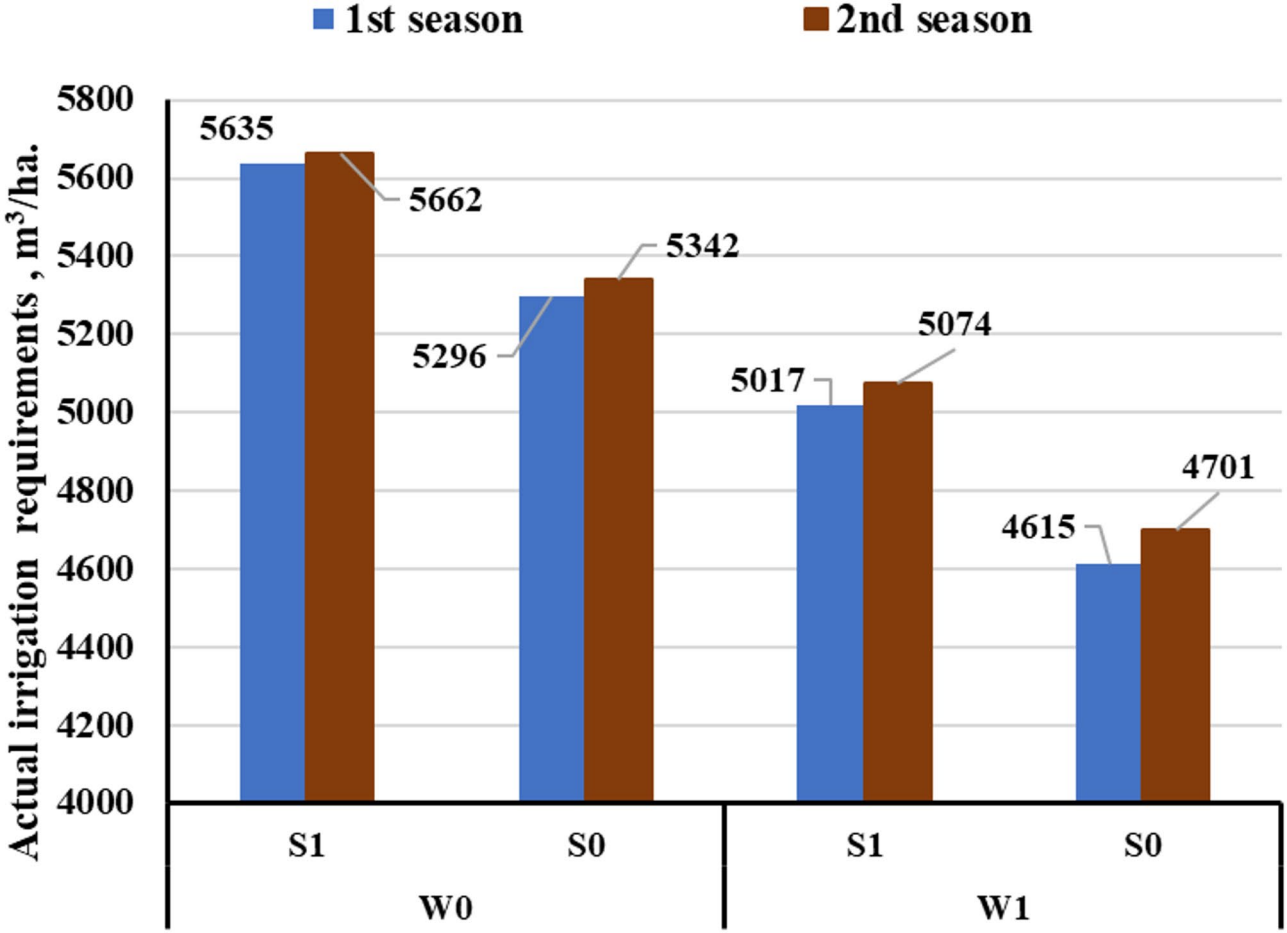
The actual irrigation requirements under different treatments.

Figure 4 illustrates the actual irrigation requirements across two growing seasons for different treatments. Irrigation requirements were determined based on soil moisture content measurements and varied among the different treatments. The available water content was higher in the magnetized water treatment compared to the other treatments. The use of magnetized water in combination with subsurface drip irrigation contributed to reducing irrigation water requirements. Accordingly, the amount of water applied for each treatment was calculated based on soil moisture measurements taken prior to irrigation. The highest value (5662 m³/ha) was recorded in the 2nd season under SDI with unmagnetized, while the lowest (4615 m³/ha) occurred in the 1 st season with magnetized and SSDI.

#### IWP

**Fig. 5:**
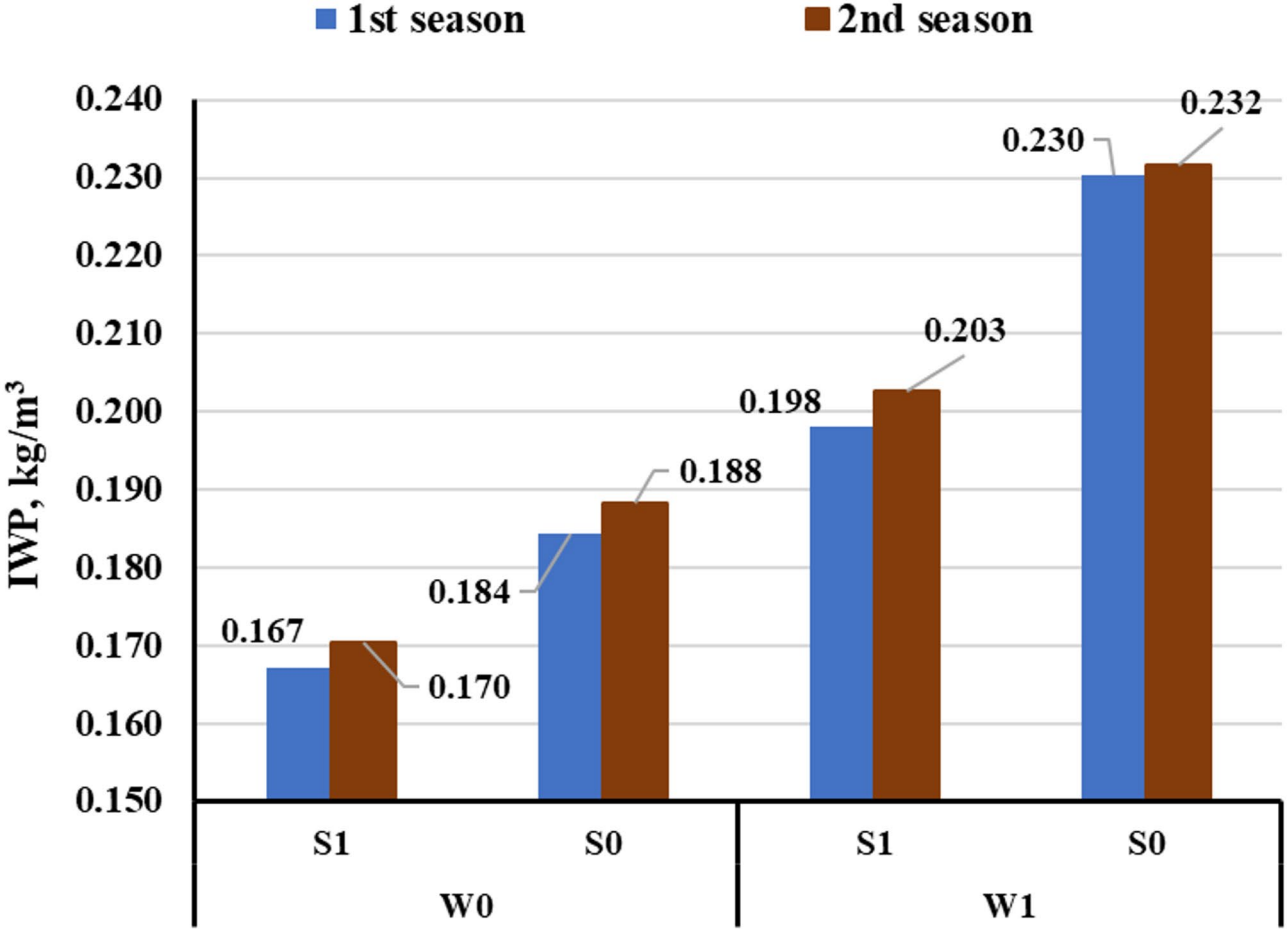
The IWP values under different treatments.

The data presented in Fig. 5 illustrate water productivity for all treatments. The use of magnetized water significantly enhanced IWP across treatments by improving water absorption and crop physiological activity. In the current study, magnetized water increased IWP by approximately 16% and 14% under subsurface drip irrigation (SSDI) in the first and second seasons, respectively, compared to non-magnetized treatments. In this study, the highest IWP values (0.230 and 0.232 kg/m³) were recorded under SSDI with magnetized water, compared to 0.184 and 0.188 kg/m³ under SDI.

### Crop characteristics

#### Growth parameters and fruit yield measurements

The results presented in Table 3 clearly demonstrate that magnetized irrigation water exerted a significant positive effect on all measured growth parameters of caraway plants compared with unmagnetized water in both seasons. The application of magnetized water increased plant height by 18.93%, accompanied by marked increases in the number of branches and umbels, suggesting that magnetic treatment enhances nutrient availability, water uptake, and overall metabolic efficiency within the plant^35,36^. When seasonal means were considered, plant height increased by 24.7% and 23.3%, the number of branches by 28.2 and 28.8%, and the number of umbels by 35.9% and 32.8% in the first and second seasons, respectively.

**Table 3:** Plant height, number of branches and number of umbels under different treatments.

Treatments		Plant height				Number of branches				Number of umbels			
		1 st season		2nd season		1 st season		2nd season		1 st season		2nd season	
Unmagnetized	**SDI**	57.67 ± 2.5		59.27 ± 3.4		33.33 ± 7.8		33.67 ± 5.5		44.00 ± 10.8		44.33 ± 6.1	
	**SSDI**	62.33 ± 6.7		63.10 ± 4.6		35.33 ± 4.7		34.67 ± 9.1		49.00 ± 4.6		50.33 ± 7.0	
Magnetized	**SDI**	70.00 ± 5.0		72.53 ± 7.0		39.00 ± 9.8		39.33 ± 3.5		57.67 ± 5.7		56.33 ± 2.5	
	**SSDI**	79.67 ± 9.6		78.40 ± 6.4		49.00 ± 7.2		48.67 ± 6.0		68.67 ± 3.8		69.33 ± 19.8	
LSD 5%													
Magnetized		22.98*		20.24**		32.73ns		14.92*		16.54**		21.55*	
Irrigation systems		7.15ns		5.29ns		6.71ns		11.06ns		12.82ns		15.60ns	
Interaction		10.11ns		7.49ns		9.48ns		15.64ns		18.14ns		22.07ns	

The irrigation technique and the use of magnetized water had a significant impact on fruit characteristics, essential oil content, and oil yield of caraway plants, as shown in Table 4. Across both seasons, magnetized water consistently enhanced fruit performance, with the weight of 1000 fruits increasing by 24% and 22.6% in the first and second seasons, respectively, and fruit yield per plant rising by 10% and 6.9% compared to unmagnetized water.

**Table 4:** Weight of 1000 fruits, Fruit yield per plant, and EO%.

Treatments		Weight of 1000 fruits (g)		Fruit yield plant (g/plant)		Essential Oil (EO)%	
		1 st season	2nd season	1 st season	2nd season	1 st season	2nd season
Unmagnetized	SDI	4.73 ± 0.78	4.81 ± 0.97	14.57 ± 0.21	15.12 ± 30.6	1.77 ± 0.04	1.78 ± 0.10
	SSDI	5.23 ± 0.38	5.37 ± 0.54	14.73 ± 0.78	15.90 ± 21.0	1.46 ± 0.11	1.49 ± 0.05
Magnetized	SDI	5.43 ± 1.06	5.51 ± 0.12	15.53 ± 0.50	16.37 ± 26.1	1.53 ± 0.07	1.45 ± 0.12
	SSDI	6.90 ± 0.87	6.97 ± 0.71	16.73 ± 0.90	16.77 ± 35.6	1.18 ± 0.06	1.23 ± 0.10
LSD 5%							
Magnetized		1.84ns	0.17*	2.76*	2.46*	0.26**	0.19**
Irrigation systems		1.45ns	1.23*	1.00ns	0.27ns	0.099**	0.19**
Interaction		2.04ns	1.74ns	1.42ns	1.01ns	0.14ns	0.27ns

**Fig. 6:**
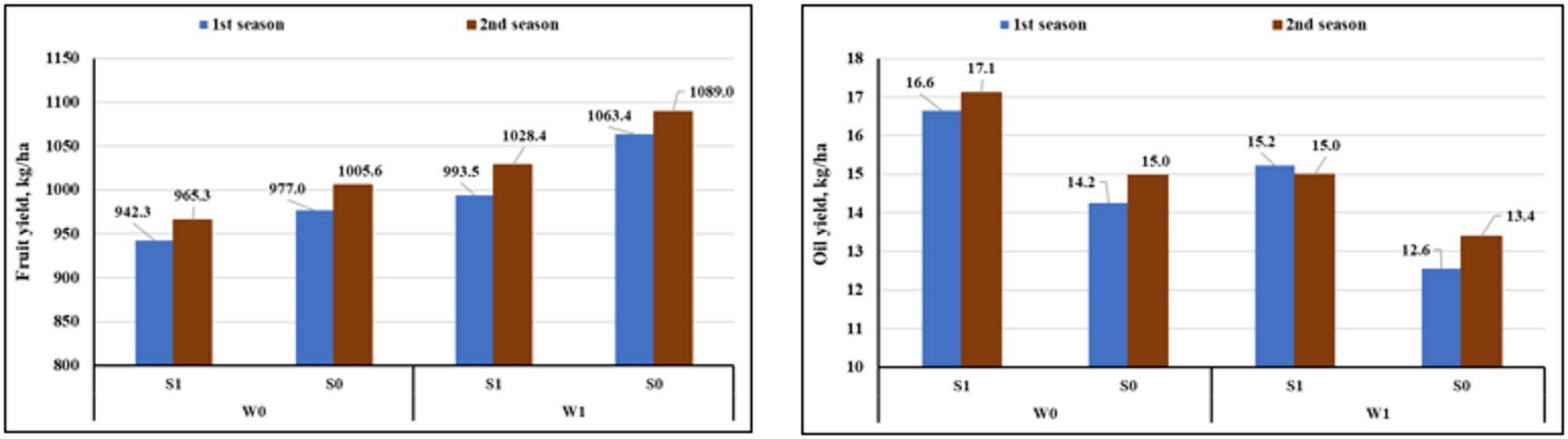
The variation in fruit and oil yield (kg/ha) under different treatments.

Moreover, as illustrated in Figure 6, magnetized subsurface irrigation also had a pronounced positive effect on overall caraway productivity per hectare. fruits yield per hectare increased by approximately 10.38% compared to the control, while essential oil yield per hectare rose by more than 24.56%, with oil yield increasing from 12.57 L/fed to 16.63 L/ha in the 1 st season and from 13.38 L/ha to 17.11 L/ha in the 2nd season. These results indicate that, despite a reduction in oil concentration, the overall oil output can be substantially increased under magnetized SSDI due to higher fruit biomass and yield.

#### Oil GC analysis

**Fig. 7:**
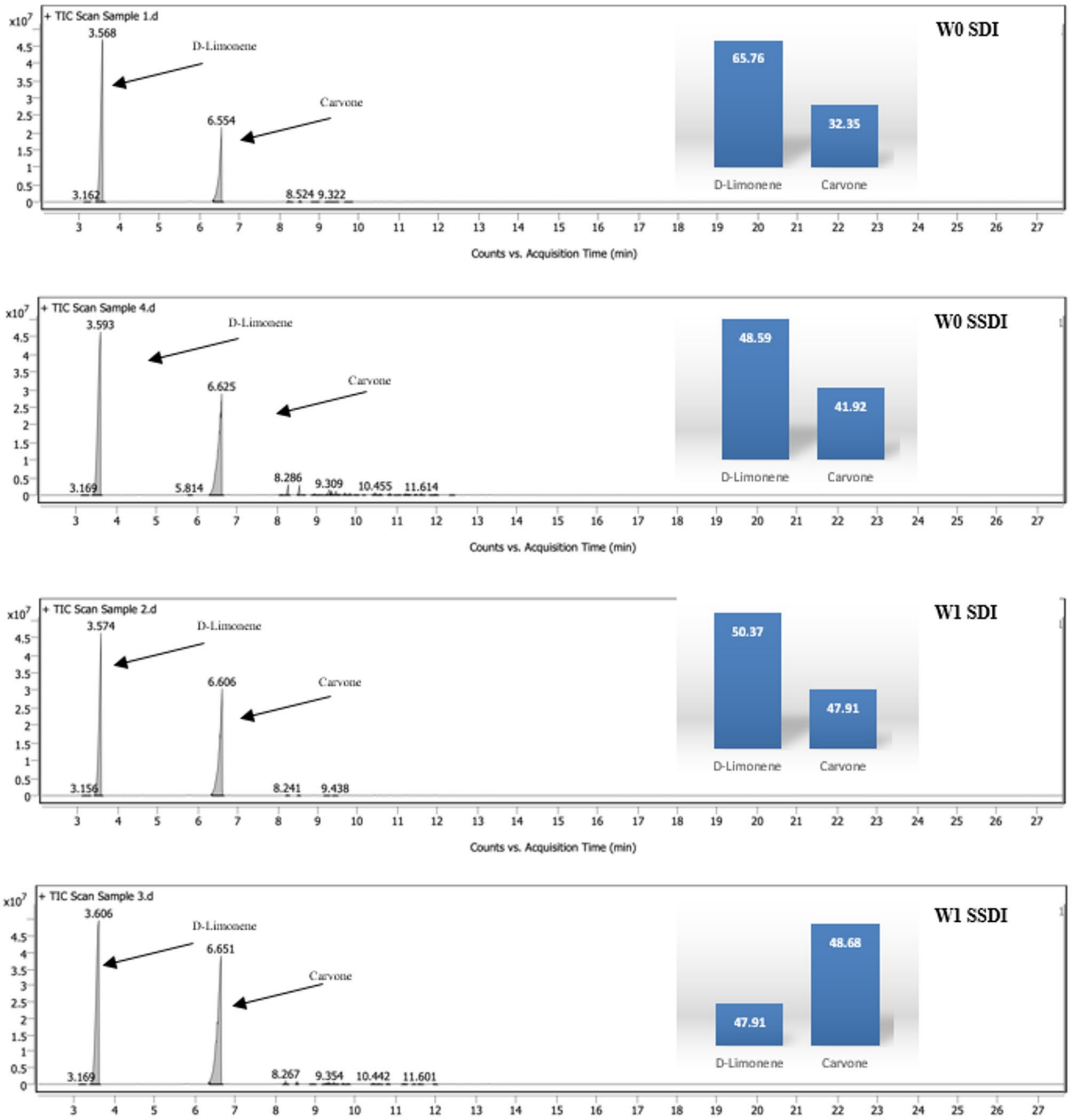
Oil GC analysis under different treatments.

Analysis of caraway oil components by GC/MS revealed that D-Limonene and Carvone were the dominant constituents across all treatments Fig. 7. Plants irrigated with unmagnetized surface water exhibited the highest D-Limonene concentration and the lowest Carvone content. In contrast, magnetized surface water significantly reduced D-Limonene from 65.76% to 50.37%, while Carvone increased from 32.35% to 47.91%. Moreover, the irrigation system had a significant effect on the composition, with subsurface irrigation enhancing Carvone concentration compared to surface irrigation. The most pronounced changes were observed under magnetized subsurface irrigation, where Carvone surpassed D-Limonene as the primary component. Previous studies have reported that Carvone and Limonene constitute approximately 95% of caraway essential oil^37,38^.

#### Economic evaluation

The economic evaluation summarized in Table 5 clearly shows that both magnetic water treatment and the type of irrigation system significantly affect production profitability. Using magnetically treated water led to higher gross and net returns compared to unmagnetized water under both surface drip irrigation (SDI) and subsurface drip irrigation (SSDI) systems. This occurred despite a slight increase in total production costs. The benefit–cost (B: C) ratio improved from 2.48 to 2.53 under SDI and from 2.54 to 2.62 under SSDI in the first season, highlighting increased economic efficiency. The combination of magnetic treatment and SSDI delivered the highest net returns (3234.12 and 3533.78$/ha) and B: C ratios (2.62 and 2.65) across both seasons, demonstrating a synergistic effect that enhances soil water conditions and crop performance. These results are consistent with previous research.

**Table 5:** Economic evaluation under different treatments.

Treatments		Total cost		Gross returns		Net returns		B: C	
		1 st season	2nd season	1 st season	2nd season	1 st season	2nd season	1 st season	2nd season
Unmagnetized	SDI	1266.34	1284.40	4410.75	4518.52	3144.42	3234.12	2.48	2.52
	SSDI	1262.87	1281.13	4469.04	4599.87	3206.16	3318.73	2.54	2.59
Magnetized	SDI	1317.23	1335.61	4650.53	4813.87	3333.31	3478.26	2.53	2.60
	SSDI	1313.13	1331.80	4751.32	4865.58	3438.19	3533.78	2.62	2.65

## Discussion

under magnetically treated surface irrigation, EC decreased from 1.042 to 0.930 dS/m (a 10.75% reduction), while under magnetically treated subsurface drip irrigation, EC declined from 0.823 to 0.670 dS/m (an 18.66% reduction). This improvement is attributed to the role of magnetic treatment in enhancing salt solubility and preventing its precipitation around the roots, thereby promoting salt leaching and minimizing accumulation within the soil profile. The best results were observed when magnetic treatment was combined with subsurface drip irrigation, highlighting the significance of this integration in improving soil properties and mitigating salinity effects on plant growth. These findings are consistent with those of Maheshwari and Grewal^35^, who reported that magnetically treated water enhances salt solubility and distribution in the soil. Similarly, Hilal et al.^17^, demonstrated that magnetic treatment improves the physical properties of water, such as reducing surface tension and increasing water infiltration into the soil, which enhances salt leaching and reduces accumulation around the root zone. Therefore, it can be concluded that magnetic water treatment mainly functions by reducing salt accumulation and improving its distribution within the soil rather than causing major changes in soil hydrogen ion concentration^39^.

The use of magnetized water consistently reduced irrigation requirements in both surface and subsurface drip systems, with reductions of 619 and 681 m³/ha, respectively. These findings are consistent with those reported by Ünlükara et al.^40^, and Ali et al.^41^. This reduction is likely attributed to the earlier crop maturity induced by magnetic treatment, as also noted by Hozayn et al.^42^, who observed enhanced physiological processes and accelerated crop development under magnetized water conditions. Additionally, SSDI lowered actual water requirements by approximately 6.02% and 8% compared to SDI under unmagnetized and magnetized conditions, respectively, due to reduced evaporation and more efficient water delivery to the root zone. These findings are consistent with Moursy et al.^43^, who reported 5.5–6.9% water savings in open fields and 5.6–6.8% under greenhouse, and with Gaafar et al.^44^, who found 5.6–7.6% reductions under different deficit irrigation levels. Similar trends were also noted by Soliman et al.^45^, and Moursy^46^, confirming the superior water-saving performance of SSDI.

The improvement in irrigation water productivity is attributed to enhanced water molecule alignment, which promotes better more efficient nutrient uptake. These results are consistent with findings by Maheshwari and Grewal^35^and Hamza et al.^39^, who reported that magnetized irrigation improved crop yield and water productivity. Moreover, the irrigation method itself played a crucial role in determining water productivity. Subsurface drip irrigation showed superior performance over SDI in terms of water savings and IWP. These results align with findings by Umair et al.^47^, and Camp et al., (1998), who reported that SSDI improved water use efficiency and yield by 20–35% compared to conventional systems. The combination of SSDI and magnetized water further enhanced crop performance and water utilization.

Magnetized irrigation water improved caraway plant growth compared with unmagnetized water. These improvements may be attributed to the accelerated activation of plant hormones and enzymes observed in plants irrigated with magnetically treated water, which promotes more efficient nutrient mobilization and transport during growth^35,36^. Consequently, magnetic treatment is likely to induce physiological and biochemical modifications within the plant system, including enhanced phytohormone synthesis, ultimately leading to increased growth and biological activity^48^. In addition to water magnetization, the irrigation method had a highly significant influence on plant performance, as subsurface drip irrigation (SSDI) consistently outperformed surface drip irrigation (SDI). This superiority was reflected in increases in plant height of 11.2 and 7.4%, number of branches of 16.6 and 14.2%, and number of umbels of 15.7 and 18.9% in the first and second seasons, respectively. The enhanced performance under SSDI can be attributed to improved soil moisture distribution within the root zone and reduced evaporative water losses, which together promote more favorable conditions for sustained plant growth^49^.

The irrigation technique and the use of magnetized water had a impact on fruit characteristics These improvements are likely attributed to accelerated hormonal and enzymatic activity during seed development, enhanced nutrient uptake, and more efficient water use^35,36^; Hozayn and Abdul Qados, 2010). Moreover, these positive effects were further amplified by subsurface drip irrigation (SSDI), which produced heavier fruit and higher fruit productivity than surface drip irrigation (SDI), likely due to improved root zone conditions, reduced evaporative losses, and more uniform soil moisture distribution^49^.

Interestingly, a negative correlation was observed between essential oil percentage and fruit production. Magnetized subsurface drip irrigation (SSDI) significantly increased fruit yield and 1000-fruit weight while reducing essential oil content, likely due to a dilution effect associated with increased biomass and accelerated growth under optimal water and nutrient conditions^50^. In contrast, caraway plants irrigated with unmagnetized surface water tended to accumulate higher essential oil content as a defense mechanism under water limitation, a trend similarly reported in Mentha sp. Marino et al.^51^, and Thymus × citriodorus “lemon thyme”^52^. Moreover, it has been suggested that increased density of oil glands under water stress conditions promotes greater accumulation of essential oils^53^.

Several factors, including genotype^54^, nutrient availability^38^, and growing conditions^55^, are known to influence the relative proportions of Carvone and Limonene in caraway fruits. Limonene-6-hydroxylase and carveol dehydrogenase are the enzymes responsible for the pathway from limonene to carvone. Under the magnetized water, the plant receives a biophysical signal that steers carbon flux away from initial growth and redirects it toward the synthesis of specific secondary metabolites, such as carvone, which act as chemical shields or signaling molecules^56^; Li et al., 2020). Under stress conditions, metabolic processes may shift to mitigate the harmful effects of reactive oxygen species (ROS) by modulating the production of specific terpenoids according to their functional roles^57^. Magnetizing water can weaken hydrogen bonds and reduce water surface tension and viscosity. It can also enhance the solubility and mobility of essential ions, thereby boosting photosynthetic efficiency and the production of metabolic energy such as Adenosine Triphosphate (ATP) and Nicotinamide Adenine Dinucleotide Phosphate (NADPH), as referenced by Cai et al.^29^, and Deng et al.^58^,. This surplus of metabolic energy allows the plant to drive the “stress-induced rerouting” of the monoterpene pathway, favoring the synthesis of oxygenated compounds (carvone) as a defensive response to transient ROS signaling^59^. Nevertheless, these changes indicate a species-specific metabolic response, reflecting a stress-induced rerouting within the carvone biosynthesis pathway. Esmaillou’s study highlights a direct correlation between the synthesis of oxygenated monoterpenes and the antioxidant enzymes including Superoxide Dismutase (SOD) enzyme, which acts as the first responder to ROS produced by irrigation stress. ROS converts superoxide radicals (O₂) into hydrogen peroxide (H₂O₂), which is then scavenged by the peroxidase enzyme^60^; Rao et al., 2025^61^. Recent data by Zhao et al.^62^, and Hasan et al.^63^, indicate that magnetized water significantly enhances POD activity to prevent lipid peroxidation.

## Conclusions

Magnetized water undergoes physical and chemical changes as it passes through a magnetic field, reducing surface tension and viscosity, enhancing water absorption, improving ion mobility, and generally optimizing soil and water properties. These changes positively affect nutrient uptake, photosynthesis, and enzyme activity, leading to improved plant growth and increased stress tolerance. The study demonstrated that the use of magnetized water significantly reduced irrigation requirements in both surface and subsurface drip systems, increased irrigation water productivity by 14–16%, and markedly enhanced caraway fruit and essential oil yields by 10.38% and over 24.56%, respectively. These findings highlight the important role of magnetic water technologies and modern irrigation practices in improving crop performance and efficiency under challenging and saline conditions, making them a promising strategy for achieving sustainable productivity.

## Data Availability

All data generated or analyzed during this study are fully included in the manuscript through its tables, figures, and result sections. No additional supplementary files are associated with this submission.
